# Schedule dependent synergy of gemcitabine and doxorubicin: Improvement of in vitro efficacy and lack of in vitro‐in vivo correlation

**DOI:** 10.1002/btm2.10082

**Published:** 2018-01-19

**Authors:** Douglas R. Vogus, Anusha Pusuluri, Renwei Chen, Samir Mitragotri

**Affiliations:** ^1^ Dept. of Chemical Engineering University of California Santa Barbara, Santa Barbara CA 93106; ^2^ John A. Paulson School of Engineering and Applied Sciences Harvard University Cambridge MA 02138; ^3^ Center for Bioengineering University of California, Santa Barbara Santa Barbara CA 93106

**Keywords:** combination chemotherapy, doxorubicin, gemcitabine, schedule, synergy

## Abstract

Combination chemotherapy is commonly used to treat late stage cancer; however, treatment is often limited by systemic toxicity. Optimizing drug ratio and schedule can improve drug combination activity and reduce dose to lower toxicity. Here, we identify gemcitabine (GEM) and doxorubicin (DOX) as a synergistic drug pair in vitro for the triple negative breast cancer cell line MDA‐MB‐231. Drug synergy and caspase activity were increased the most by exposing cells to GEM prior to DOX in vitro. While the combination was more effective than the single drugs at inhibiting MDA‐MB‐231 growth in vivo, the clear schedule dependence observed in vitro was not observed in vivo. Differences in drug exposure and cellular behavior in vivo compared to in vitro are likely responsible. This study emphasizes the importance in understanding how schedule impacts drug synergy and the need to develop more advanced strategies to translate synergy to the clinic.

## 1 INTRODUCTION

Combination chemotherapy is commonly employed as front‐line therapy in many advanced malignancies.^1^, ^2^, ^3^, ^4^, ^5^ Chemotherapeutic agents are combined to overcome various mechanisms of single drug resistance and to enhance antitumor activity compared to single drugs. While drug combinations are commonly selected by combining drugs with different mechanisms of dose‐limiting toxicity, combination therapy is often only marginally more effective at reducing tumor burden while inducing more toxicity compared to single drug therapy.^6^ When establishing how the drugs are administered in combination, dosing regimens are typically based upon the drug administration schedules and maximum tolerated doses (MTDs) determined during phase I trials for the individual drugs.^7^ The dose of each drug in combination is administered as close to the single drug MTD as possible, which does not necessarily result in the highest therapeutic index possible for the given drug pair.

To reduce systemic toxicity, synergistic drugs or drugs that work favorably together, are combined in hopes of lowering the required dose to achieve a therapeutic effect. In the past few decades, there have been considerable efforts in developing methods to empirically identify synergistic drug pairs in vitro.^8^, ^9^, ^10^, ^11^ Many in vitro studies have shown that drug synergy is dependent on how the drugs are combined, such as the molar ratio of the drugs in the combination; therefore, precision is required in translating these synergistic drug pairs effectively to the clinic.^11^

While often neglected, drug schedule plays a significant role in the efficacy of a given combination drug pair. For example, many in vitro studies have shown that sequentially staggering chemotherapeutic agents in vitro can increase drug combination activity and synergy.^12^, ^13^, ^14^, ^15^ Often times, temporally staggering drugs can increase levels of apoptotic activity^16^, ^17^, ^18^ or induce cell cycle mediated effects^19^, ^20^ which result in greater cell death. Although there are many studies which study the mechanisms that are responsible for schedule dependent synergy, few studies also determine if this synergy translates to an in vivo model. Furthermore, even fewer studies directly compare different combination drug schedules in vivo, to evaluate if the optimal schedule identified with in vitro studies is also applicable in the in vivo environment.

Improving combination chemotherapeutic regimens is particularly important in triple negative breast cancer, as cells do not over‐express the common receptors (progesterone (PR), estrogen (ER), and human epidermal growth factor (HER2)) which are the common therapeutic targets for other breast cancer types.^1^, ^21^ While triple negative breast cancer patients are typically responsive to chemotherapy, patients with metastasized triple negative breast cancer have poor prognosis. Many combination regimens have been evaluated in the clinic to treat these difficult cases; however, toxicity has limited the utility of the combination therapies.^1^

Here, using the triple negative breast cancer cell line MDA‐MB‐231 as a model cell line, we study the impact of drug schedule on the activity of chemotherapeutic combinations in vitro and in vivo. We first identify a synergistic drug pair from a panel of FDA approved drugs, and then show that drug schedule governs the activity of the drug combination in vitro. We further show that the drug combination is effective at treating tumor‐bearing mice; however, further work is required to optimize the synergy in vivo.

## 2 MATERIALS AND METHODS

### 2.1 Cell culture

Cells were maintained in their proper media supplemented with 50 I.U./ml penicillin and 50 μg/ml streptomycin (Thermo Fisher Scientific) in a humidified incubator at 37°C and 5% CO_2_. MDA‐MB‐231 human breast cells (ATCC) were cultured in RPMI‐1640 media (Thermo Fisher Scientific) supplemented with 10% fetal bovine serum. MCF‐10a human nontumorigenic breast epithelial cells (ATCC) were cultured in MEBM media kit supplemented with hydrocortisone, hEGF, insulin, and bovine pituitary extract (Lonza) in addition to 100 ng/ml cholera toxin.

### 2.2 Cell viability assays

Cell viability was measured by MTT assay after exposure to various chemotherapeutic agents (LC Laboratories). Cells (5 x 10^3^ MDA‐MB‐231 or 1 x 10^4^ MCF‐10a) were allowed to adhere overnight in 96‐well plates. Cells were then incubated with single drugs or drug combinations for 72 hr (unless specified otherwise). When sequentially exposing drugs to cells, the first drug was aspirated and replaced with the second drug in media by pipetting along the side of the wells to avoid physically disrupting the cells. After drug incubation, cells were incubated with media containing 0.5 mg/ml MTT (Thermo Fisher Scientific) for 3.5–4 hr. After replacing the MTT solution with DMSO, the plate was shaken for 30 min to solubilize the formazan crystals, and the absorbance of each well was measured at 570 nm (Tecan Infinite M200 Pro). Fractional cell inhibition was then calculated by subtracting the viability of the treated cells from the viability of the control cells, normalized by the viability of the control cells.

Fractional cell inhibition was measured after exposure to drugs at a range of concentrations to generate dose‐response curves using the median effect model.^22^ The dose response curves were used to calculate IC_50_ values and the combination index (CI), which determines synergy of drug combinations.^22^ Error bars for the CI were calculated by propagating the confidence intervals from the individual drug fits and the error in toxicity for the combination treatment. When calculating the CI, single drug dose response curves were used with the identical schedule as given in the combination treatment.

### 2.3 Cell cycle distribution

Cell cycle distribution of MDA‐MB‐231 cells was measured with flow cytometry using the nuclear stain propidium iodide (PI) (Sigma Aldrich). Briefly, cells (7.5 x 10^5^) were seeded into T25 flasks and allowed to adhere overnight. After incubating cells with GEM and DOX solutions for 24–72 hr, adherent cells were collected with trypsin and washed 2x in PBS (5 ml). Cells were then fixed by slowing adding 70% (v/v) ethanol (5 ml) into a concentrated cell suspension (0.5 ml). After incubating the cells in ethanol for at least 1 day at 4°C, the cells were washed 2x in PBS through repeated centrifugation steps. The cells were then stained in a PBS solution (0.5 ml) containing Tween 20 (0.01%, v/v), 100 µg/ml of RNAase A (Sigma Aldrich), and PI (10 µg/ml) for 30 min at room temperature in the dark. After staining, cells were kept on ice and fluorescence was quantified using a Becton Dickinson FACSAria cell sorter with a 633‐nm laser with 610/20 PMT.

### 2.4 Caspase 3 fluorometric assay

Induction of apoptosis was determined by measuring caspase 3 activity using the EnzChek caspase 3 assay kit (Thermo Fisher Scientific) according to manufacturer's instructions. Briefly, cells were seeded into T25 flasks (7.5 ml; 1 x 10^5^ cells/ml) and allowed to adhere overnight. Following incubation with drug formulations, both floating and adherent cells were washed with PBS and lysed. Cell lysates were then centrifuged at 5,000 rpm for 5 min and the supernatant was collected. Caspase 3 activity was quantified by incubating 50 µl of the supernatant with 50 µl of the 2× working solution containing the substrate Z‐DEVD‐AMC for 30 min at 37°C. The fluorescence intensity was then measured of each well at an excitation/emission of 342/441 nm (Tecan Plate Reader). Caspase 3 activity was calculated with respect to an AMC standard curve.

Caspase activity was then normalized to the total protein content, using the micro BCA assay (Thermo Scientific). Briefly, 15 µl of the leftover supernatant was diluted with 135 µl of DI water and incubated with 150 µl of the BCA solution for 1.5 hr at 37°C. After incubation, protein content was determined by reading the absorbance at 562 nm (Tecan Plate Reader) with internal protein standards of bovine serum albumin.

### 2.5 In vivo MDA‐MB‐231 xenograft model

An MDA‐MB‐231 xenograft model was used to evaluate the efficacy of DOX and GEM combination therapy in athymic nude mice (Charles Rivers Laboratories). All experiments were performed according to approved protocols by the Institutional Animal Care and Use Committee of the University of California, Santa Barbara. MDA‐MB‐231 cells (2.5 x 10^6^), suspended into 100 µl of 1:1 PBS : Matrigel (Corning), were injected subcutaneously into the inguinal mammary fat pad of 6–8 week old athymic, nude mice. Starting 11 days after tumor transplantation, when tumors reached 50 mm^3^, mice were treated either once or once a week for 4 weeks. All drug formulations were prepared in sterile saline (0.9% (wt/vol) NaCl). Tumor volume was measured with a caliper using the following equation: $V=\frac{L{*W}^{2}}{2}$, where is the *L* is the longest dimension of the tumor and *W* is the shortest.

## 3 RESULTS

### 3.1 Identification of synergistic chemotherapeutic drug pairs

**Figure 1:**
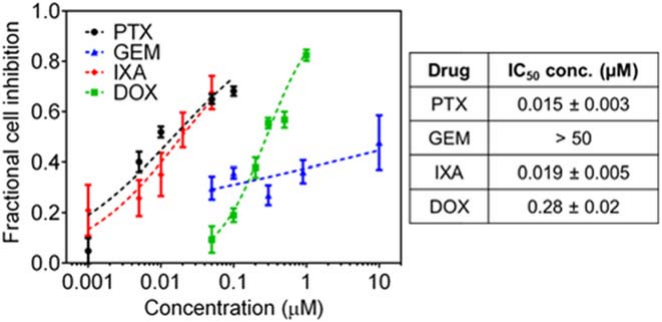
Growth inhibition of MDA‐MB‐231 cells after 72 hr of single drug exposure. Markers represent experimental data and curves represent best‐fit median effect model. Error bars represent 95% CI (*n* ≥ 12 wells)

To identify synergistic drug pairs, MDA‐MB‐231 cells were exposed to a panel of FDA approved chemotherapeutic agents with different mechanisms of action, including doxorubicin (DOX), paclitaxel (PTX), ixabepilone (IXA), and gemcitabine (GEM). Cell viability was measured after exposure to single drugs using the MTT assay to generate dose response curves and determine IC_50_ concentrations (Figure 1). Cells were responsive to both mitotic inhibitors PTX and IXA, with IC_50_ concentrations of 19 ± 5 nM and 15 ± 5 nM, respectively, and to DOX, with an IC_50_ concentration of 0.28 ± 0.02 µM. Cell growth was inhibited by GEM at low concentrations (nM range); however, high GEM concentrations (>1 µM) were ineffective at killing more cells, resulting in a very large IC_50_ concentration.

**Figure 2:**
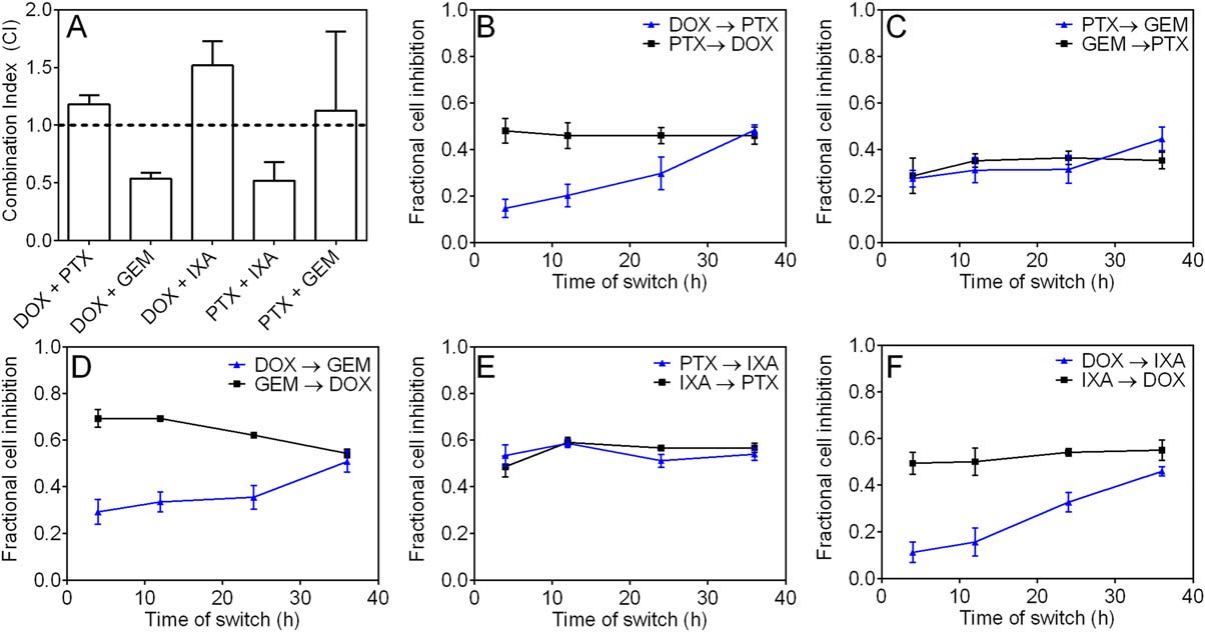
MDA‐MB‐231 cell viability and synergy after 72 hr exposure to combinations of DOX (0.3 µM), PTX (0.005 µM), GEM (0.2 µM), and IXA (0.015 µM). (A) Combination index of MDA‐MB‐231 cells after concurrent exposure to drug combinations. (B–F) Fractional cell inhibition of MDA‐MB‐231 cells after the sequential exposure of drug combinations. The first drug was aspirated and replaced with the second drug at the specified time points and cell viability was measured at 72 hr. Error bars represent 95% CI (*n* ≥ 12 wells)

After establishing the IC_50_ concentration of each drug, drugs were tested in combination. First, cells were exposed concurrently to a combination of drugs at single drug concentrations between the respective IC_25_ and IC_50_ concentrations. This concentration range was used to ensure that the drug concentration was high enough to impact cell viability without one drug inhibiting all cell growth. The combinations of GEM/DOX and IXA/PTX were synergistic at inhibiting tumor cell growth, with CI values less than 1 (Figure 2A).

MDA‐MB‐231 cells were then sequentially exposed to the same drug pairs, to determine if drug administration schedule impacts cellular viability (Figure 2B–E). For example, Drug A was given to the cells for 4, 12, 24, or 36 hr and then replaced with Drug B for the remaining time in the 72 hr drug exposure. The combination of GEM and DOX, which was synergistic with a CI of 0.53 ± 0.05 when given concurrently, was significantly more toxic when cells were exposed to GEM prior to DOX compared to the reverse schedule. Furthermore, the combinations of DOX with the two mitotic inhibitors (PTX and IXA) were more toxic when the mitotic inhibitor was given prior to DOX. The toxicity of PTX and IXA showed minimal schedule dependence on MDA‐MB‐231 cells.

The changes in cellular viability after exposing cells to different combination schedules result from both changes in drug–drug interactions and changes in the exposure time of the individual drugs with different pharmacodynamic properties. For example, the 72 hr IC_50_ of DOX is 0.28 ± 0.02 µM, and the IC_50_ is increased to 0.59 ± 0.05 or 2.2 ± 0.4 µM if the cells are exposed to DOX for only the first 24 or 4 hr, respectively (Supporting Information Figure S1). Furthermore, the IC_50_ is increased to 0.42 ± 0.03 or 1.6 ± 0.3 µM, if exposure to DOX is delayed by 4 or 24 hr, respectively. Therefore, to understand the impact that drug schedule has on drug–drug interactions, drug synergy must be evaluated using dose response curves for the single drugs, at identical exposure schedules which were given in the combination treatment, as previously done by Chou.^13^

### 3.2 Schedule dependent synergy between GEM and DOX

**Figure 3:**
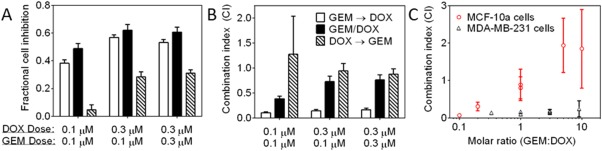
(A) Cell viability of MDA‐MB‐231 cells after 72 hr exposure to DOX and GEM combinations. (B) Drug synergy for each drug treatment shown in (A). Drug exposure schedules included concurrent exposure to GEM/DOX (72 hr) and the sequential exposure to GEM (24 hr) → DOX (48 hr) and DOX (24 hr) → GEM (48 hr). Legend for (A,B) is shown in (B). (C) Synergy at inhibiting the growth of MDA‐MB‐231 and MCF‐10a cells after exposure to GEM (24 hr) → DOX (48 hr) at different GEM : DOX ratios. Drug concentrations were selected near the IC_50_ for each cell line. Data represents mean ± 95% confidence intervals (*n* ≥ 12)

Because GEM and DOX were both synergistic when given concurrently and showed a strong schedule dependence, this drug pair was studied in detail. MDA‐MB‐231 cell viability was measured after incubating cells with different sequences of GEM and DOX at different drug doses (Figure 3A). Sequentially exposing the cells to GEM → DOX or concurrently to GEM and DOX inhibited more cell growth than exposing the cells to DOX → GEM at each drug dose tested. Combination indices were calculated for each GEM and DOX combination (Figure 3A) based on single drug dose response curves (Supporting Information Figure S1), which were made with identical exposure schedules compared to the combination treatments. It was more synergistic (lower CI) to incubate the cells with GEM prior to DOX than to expose the cells to GEM and DOX simultaneously or to DOX prior to GEM.

In addition to the triple negative breast cancer cell line (MDA‐MB‐231), the nontumorigenic breast epithelial cell line MCF‐10a, which served as a control, was exposed to the schedule of GEM (24 hr) → DOX (48 hr). The effect of molar ratio on drug synergy is shown for each cell line in Figure 3B. The schedule of GEM → DOX was synergistic on MCF‐10a cells for GEM : DOX molar ratio less than one; however, it was antagonistic for GEM : DOX molar ratios greater than one. On the contrary, the schedule of GEM : DOX was extremely synergistic (CI < 0.2) on the triple negative breast cancer cells at all of the molar ratios tested.

### 3.3 Caspase activity after exposure to GEM and DOX

**Figure 4:**
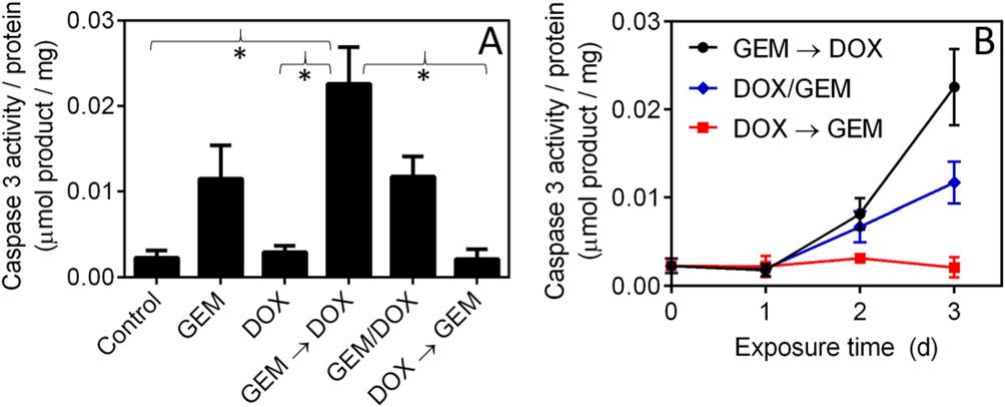
(A) Caspase 3 activity after 72 hr exposure to GEM (0.3 µM), DOX (0.3 µM), and GEM and DOX combinations. Cells were exposed to GEM for 1 day then DOX for 2 days, GEM and DOX for 3 days, or DOX for 1 day then GEM for 2 days for the schedules of GEM → DOX, GEM/DOX, and DOX → GEM, respectively. Statistical significance (*p < .05) performed by two‐tailed Students *t*‐test. (B) Caspase 3 activity measured during exposure to different combinations of GEM and DOX described in (A). Data represents mean ± SEM (*n* = 3)

Caspase 3 activity was measured after incubating cells with different GEM and DOX schedules to evaluate the effect of drug schedule on the induction of apoptosis (Figure 4). Exposing MDA‐MB‐231 cells to GEM prior to DOX increased caspase 3 activity 2x and 10x compared to exposing the cells to GEM and DOX concurrently and to DOX prior to GEM, respectively. Compared to GEM, the drug combination of GEM and DOX only increased caspase activity if GEM was given prior to DOX.

### 3.4 Cell cycle dependent interactions between GEM and DOX

**Figure 5:**
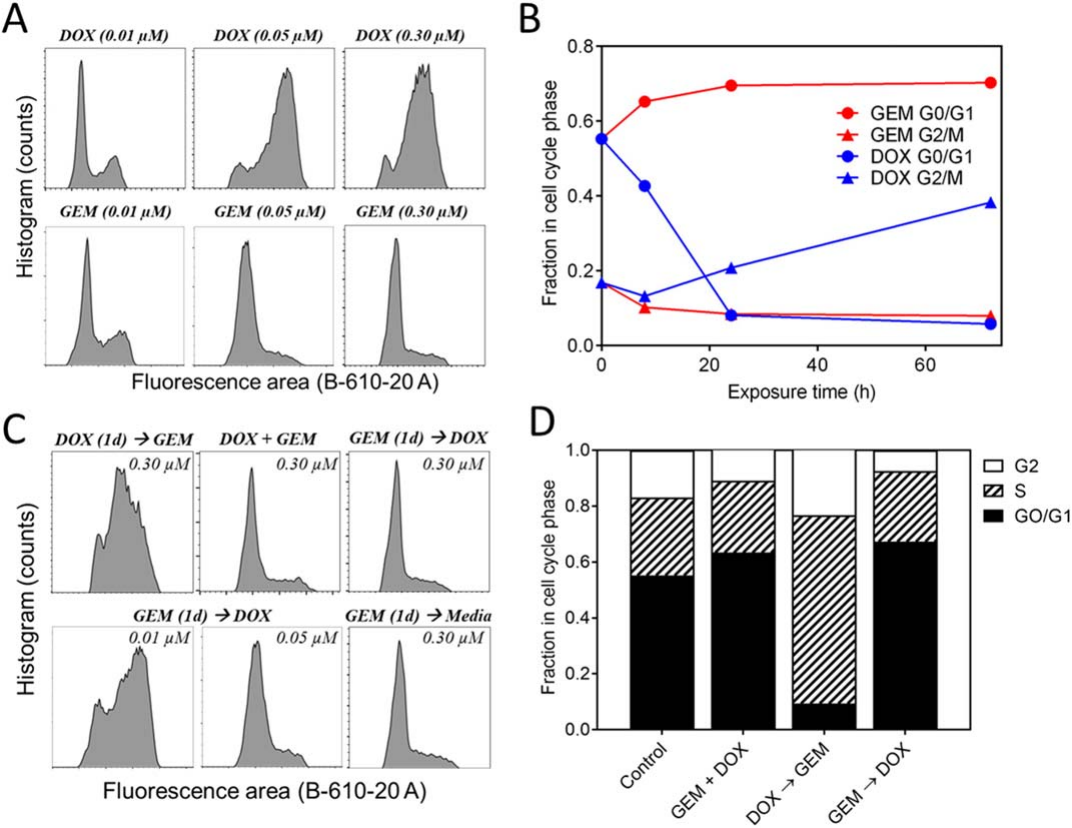
Cell cycle distribution of MDA‐MB‐231 cells after exposure to GEM, DOX, and GEM/DOX combinations. (A) Representative flow cytometry histograms after exposure to GEM or DOX for 72 hr at indicated concentrations. (B) Progression of cell cycle phases after exposure to GEM (0.3 µM) or DOX (0.3 µM). (C) Representative flow cytometry histograms after exposure to GEM/DOX combinations (equimolar concentrations) for 72 hr. (D) Fraction of cells in each phase of the cell cycle after exposure to GEM/DOX combinations (both at 0.3 µM) for 72 hr

GEM and DOX are known to be highly active in specific stages of the cell cycle. Therefore, to gain mechanistic insight on why GEM → DOX was more effective at inhibiting cancer cell growth than other schedules, cell cycle distribution was measured by incubating cells with the nuclear stain PI after drug exposure (Figure 5). MDA‐MB‐231 cells arrested in the S phase after exposure to 10 nM GEM and in the G0/G1/early S phases after exposure to 50 and 300 nM GEM. Cells accumulated in the S phase and G2/M phases after exposure to 10 nM and 50, 300 nM DOX, respectively. After exposing the cells to a high GEM dose (300 nM), cells accumulated in the G0/G1/early S phases within 24 hr. On the contrary, it took approximately 24–72 hr for cells to completely accumulate in the G2/M phases after exposure to DOX (300 nM). GEM was also washed out with media after 24 hr exposure, and the cell cycle distribution remained arrested 48 hr later (Figure 5C).

After incubating the cells with DOX/GEM combinations, the resulting cell cycle distribution matched closely to that of which the cells were exposed to first. For example, cells accumulated in the G0/G1/early S phases and G2/M phases after exposure to GEM → DOX and DOX → GEM, respectively (Figure 5C,D). Interestingly, after the concurrent exposure to GEM/DOX, cells also accumulated in the G0/G1/early S phases. While cells still accumulated in the G0/G1/early S phases after exposure to GEM → DOX at 50 nM, cells accumulated in the S phase after exposure to GEM → DOX at 10 nM.

### 3.5 Evaluation of GEM and DOX synergistic effect in tumor bearing mice

**Figure 6:**
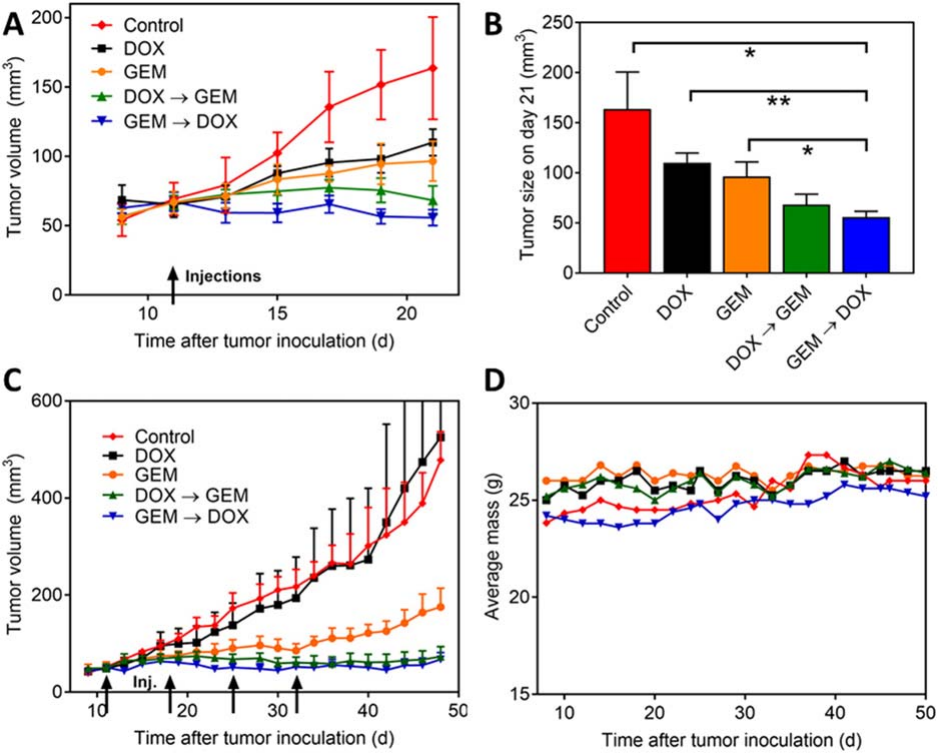
Inhibition of tumor growth using DOX and GEM. Mice bearing orthotopic, MDA‐MB‐231 xenografts were treated by DOX, GEM, or DOX/GEM. All DOX (2 mg/kg) and GEM (20 mg/kg) doses were consistent for both studies and all experimental groups. The GEM → DOX and DOX → GEM groups have a 1‐day delay between the injections of each drug. (A) Tumor growth inhibition with one i.v. injection starting on day 11. (*n* = 5) (B) Final tumor volume for the study in (A). Statistical significance (*p < .05, **p < .01) performed by two‐tailed Students *t*‐test. (C) Tumor growth inhibition with 4 x 1w i.v. injections starting on day 11 (DOX and GEM groups: *n* = 4, DOX → GEM, GEM → DOX, and control groups: *n* = 5). (D) Average body masses throughout the course of injections shown in (C). All error bars represent SEM

To evaluate if the synergistic effect of DOX and GEM in vitro translates to improved efficacy in vivo, the free drugs were used to treat mice bearing orthotopic, MDA‐MB‐231 xenografts. In the first study, mice received one cycle of chemotherapy consisting of either DOX, GEM, DOX → GEM, or GEM → DOX. Using low doses of both DOX (2 mg/kg) and GEM (20 mg/kg), the schedule of GEM → DOX inhibited tumor growth up to 20 days post treatment, which was significantly more effective than DOX or GEM on its own (Figure 6A,B). The schedule of GEM → DOX (which was determined to be most potent in vitro) was only marginally more effective than the reverse schedule of DOX → GEM.

Next, 4 weekly injections of chemotherapy were given to attempt to eradicate the tumors. Again, the combinations of GEM → DOX and DOX → GEM were significantly more effective at inhibiting tumor growth than the individual drugs (Figure 6C). Both schedules of DOX and GEM prevented tumor growth up to approximately 45 days. It is important to note that the repeated injections of the low doses of GEM and DOX caused no observable toxicity as evident by no change in body weight throughout the course of the study (Figure 6D).

## 4 DISCUSSION

There have been extensive efforts to identify synergistic chemotherapeutic agents to improve the efficacy of chemotherapy. While many studies have identified synergistic drug pairs for specific cancer cells in vitro, few report if the synergy offers improved therapeutic benefits in vivo. Here, we specifically studied the impact that drug schedule has on synergy in vitro, and if optimal drug schedules identified in vitro can be used to more effectively treat tumor‐bearing mice in vivo.

To identify a synergistic drug pair, four FDA approved chemotherapeutic agents were screened to treat MDA‐MB‐231 cells. The dependence of synergy on schedule was studied in detail for DOX and GEM, which was synergistic when given concurrently and demonstrated a strong schedule dependence on cell growth inhibition. Previous clinical trials have shown benefits to combining DOX and GEM while treating advanced and metastatic breast cancer patients; however, neutropenia was often reported as limiting the administered dose.^23^, ^24^, ^25^, ^26^ Therefore, understanding how to improve the potency of GEM and DOX at lower drug doses is of interest.

Giving GEM prior to DOX was significantly more synergistic than giving the two drugs concurrently or in the reverse sequence. The increase in synergy upon giving GEM prior to DOX was consistent with an increase in caspase activity (Figure 4). Previous reports have also shown that the schedule in which cancer cells are exposed to chemotherapeutic drugs can significantly impact the induction of pro‐apoptotic pathways.^16^ Furthermore, the advantages of exposing cells in vitro to GEM prior to a topoisomerase inhibitor have previously been reported.^27^ Interestingly, the schedule of GEM (1 day) → DOX (2 day) was not synergistic on immortalized, nontumorigenic breast epithelial cells (MCF‐10a) if GEM was given in molar excess of DOX. This provides evidence that by manipulating the schedule and ratio in which cells are exposed to DOX and GEM, it is possible to selectively induce higher toxicity toward breast cancer cells (MDA‐MB‐231) as compared to immortalized, nontumorigenic breast cells (MCF‐10a).

While exposing MDA‐MB‐231 cells to GEM prior to DOX was most effective at inhibiting cell growth, it was challenging to determine the limitations on timing and drug concentration to achieve synergy from in vitro toxicity alone. Analyzing cell cycle distribution provided a useful tool to understand how the cells responded to different drug doses and schedules, as both DOX and GEM are known to induce cell cycle arrest and be more active in the early S phase of the cell cycle.^28^, ^29^ After exposing MDA‐MB‐231 cells to a high concentration of GEM (≥50 nM), cells arrested in the G0/G1/early S phases, while they arrested in the G2/M phases after exposure to DOX (≥50 nM) (Figure 5A), consistent with previous reports.^30^, ^31^ Therefore, it is not surprising that DOX → GEM was ineffective because GEM is most effective in the S phase. When DOX and GEM were given concurrently at the same dose, cells accumulated in the G0/G1/early S phases, which may explain why concurrent exposure was still synergistic.

Cell cycle distribution was dependent upon both concentration and exposure time, indicating that there are limitations on drug concentrations and schedules to achieve synergy. Cells accumulated quickly (<24 hr) in the G0/G1/early S phases after exposure to GEM, while it took longer (24–72 hr) to accumulate in the G2/M phases after exposure to DOX. Furthermore, low drug concentrations (<50 nM) which did not induce any cytotoxic effects over 72 hr, did not induce cell cycle arrest in the same manner as higher drug concentrations. For example, while cells accumulated in the G0/G1/early S phases after exposure to GEM → DOX at a concentration of 50 nM (similar to 300 nM), cells accumulated in the S phase at a concentration of 10 nM (Figure 5C) consistent with 10 nM GEM (Figure 5A). Interestingly, 48 hr after a 24 hr GEM exposure (0.30 µM) and a media washout, cells remained locked in the G0/G1/early S phases, suggesting that DOX may be administered significantly after GEM to still achieve synergy; however, this was not studied in detail.

The in vitro data showed that DOX and GEM are synergistic at inhibiting the growth of MDA‐MB‐231 cells and the synergy is enhanced by giving GEM prior to DOX. Therefore, the potential benefit of combining GEM and DOX with different administration sequences was evaluated in vivo. Low doses of DOX (2 mg/kg) and GEM (20 mg/kg) were selected based on previous studies to minimize systemic toxicity, while still being therapeutically active.^32^, ^33^, ^34^, ^35^ The drug doses do not reflect the relative drug concentrations that the tumor cells were exposed to in vivo because GEM is cleared much faster than DOX. While DOX has an elimination half‐life of approximately 10 hr in mice,^36^ GEM is quickly deaminated to the inactive metabolite 2′,2′‐difluorouridine (dFdU) by cytidine deaminase resulting in an elimination half‐life of approximately 30 min.^37^

Repeated injections of DOX (2 mg/kg) were ineffective at inhibiting MDA‐MB‐231 xenograft growth, consistent with previous reports which used higher cumulative DOX doses.^32^, ^33^ On the contrary, repeated injections of GEM (20 mg/kg) were moderately effective at inhibiting MDA‐MB‐231 tumor growth in vivo, consistent with previous studies using similar doses.^34^, ^35^ The relative efficacy in vivo of each individual drug is contradictory to the in vitro toxicity. The 72 hr IC_50_ of DOX (0.28 µM) in vitro was significantly lower than GEM (>10 µM); however, GEM was much more effective than DOX at inhibiting tumor growth in vivo. Although GEM was given at a higher dose than DOX, 20 mg/kg compared to 2 mg/kg, GEM is cleared faster than DOX.^36^, ^37^

The drugs were also administered sequentially in combination (GEM → (1 day) DOX and DOX → (1 day) GEM) for one injection and four repeated injections. Both combination drug regimens were more effective at inhibiting tumor growth than the individual drugs on their own. In fact, four repeated cycles of both drug combination regimens stopped tumor growth 40 days after the first injection. In combination, the cumulative dose of both DOX and GEM is much lower than the single drug doses required to inhibit tumor growth to the same extent.^32^, ^33^, ^34^ Interestingly, the sequential administration of DOX → GEM (1 cycle or 4 cycles) is essentially just as effective as the sequential administration of GEM → DOX, contradictory with the in vitro cell viability and apoptosis assays.

Previous studies have shown that optimal drug exposure schedules identified in vitro can offer improved therapeutic benefits in vivo^16^, ^38^, ^39^; however, we demonstrate here that it can be nontrivial to translate optimal drug schedules in vivo. The difference in how drug schedule impacts GEM and DOX activity in vivo compared to in vitro is interesting and is likely due to differences in the tumor environment and drug exposure. In vivo, the cancer cells will likely develop different characteristics and respond to the drug combination differently, due to the complex tumor microenvironment consisting of stromal and immune cells.^40^, ^41^, ^42^ In addition, tumor growth in response to chemotherapy can be impacted by the immune response in vivo^43^; however, the impact of drug combination schedule on the activation of the immune system was not accounted for in the in vitro studies. Many advanced in vitro models have been developed, which attempt to replicate the complex tumor environment, and they should be used in future studies to further study the impact of drug schedule in combination therapies.^44^

Another significant challenge with regards to optimizing schedule in combination therapies, is the fast and variable clearance of each drug in vivo. For example, here, GEM and DOX are cleared with elimination half‐lives of 30 min and 10 hr, respectively.^36^, ^37^ The impact of these pharmacokinetic parameters on the in vitro conclusions about drug synergy is unknown and challenging to accurately study in vitro. To take full advantage of the benefits of combining multiple chemotherapeutics, it is necessary to precisely control how cells are exposed to the drugs in vivo.

The development of combination delivery platforms has made it possible to control the relative concentration and exposure sequence of multiple drugs in vivo. In fact, various delivery systems have already been engineered to deliver DOX and GEM simultaneously in the same carrier.^45^, ^46^, ^47^, ^48^, ^49^ By delivering multiple therapeutics in a single vehicle, drug ratios, and release kinetics can be finely controlled to more closely mimic optimal drug conditions discovered in vitro.^50^, ^51^ As the field continues to develop, an emphasis needs to be placed on translating synergistic drug interactions by optimizing drug ratio and release rates from these delivery systems.

The challenge in being able to predict the activity of a drug combination in vivo on a single cell line, which was studied extensively in vitro, reiterates the difficulty in improving combination chemotherapy regimens in human patients. Future improvements in combination therapies will not only depend on the ability to improve translation from in vitro assays to animal models, but also on the translation from animal models to human patients. The development of tools to quickly study the response of patient tumors to combination treatments can help improve the optimization of drug combinations.^52^, ^53^ Moving forward, it is critical to simultaneously study the impact of drug schedule on tumor regression in multiple tumor phenotypes, while also studying the impact of drug schedule on off‐site toxicity.

## 5 CONCLUSIONS

The use of synergistic drug pairs can improve the efficacy of combination chemotherapy by lowering required drug doses and reducing toxicity; however, synergistic drug interactions often require very specific conditions. The impact of drug schedule on synergy for DOX and GEM was studied in detail on the triple negative breast cancer cell line MDA‐MB‐231. Sequentially exposing the cells to GEM to DOX induces more apoptosis and is more synergistic than concurrent drug exposure or the reverse sequence. While the exact mechanism of drug synergy is unclear, changing drug schedule significantly changes resulting cell cycle distribution. The combination of DOX and GEM is effective at treating an MDA‐MB‐231 xenograft in vivo; however, changing drug administration sequence does not impact tumor regression. Developing more predictive in vitro models and simple combinatorial delivery vehicles should help translate drug synergy to the clinic.

## Supporting information

Additional Supporting Information may be found online in the supporting information tab for this article.

Supporting Figure
