# Supplementary material for: Schedule dependent synergy of gemcitabine and doxorubicin: Improvement of in vitro efficacy and lack of in vitro‐in vivo correlation
Source: Bioeng Transl Med. 2018 Jan 19;3(1):49–57. doi: 10.1002/btm2.10082 (PMC5773969; doi:10.1002/btm2.10082)
Supplement: Supplementary file 1 — Supporting Figure [file BTM2-3-49-s001.docx]

**Supplementary Figures**


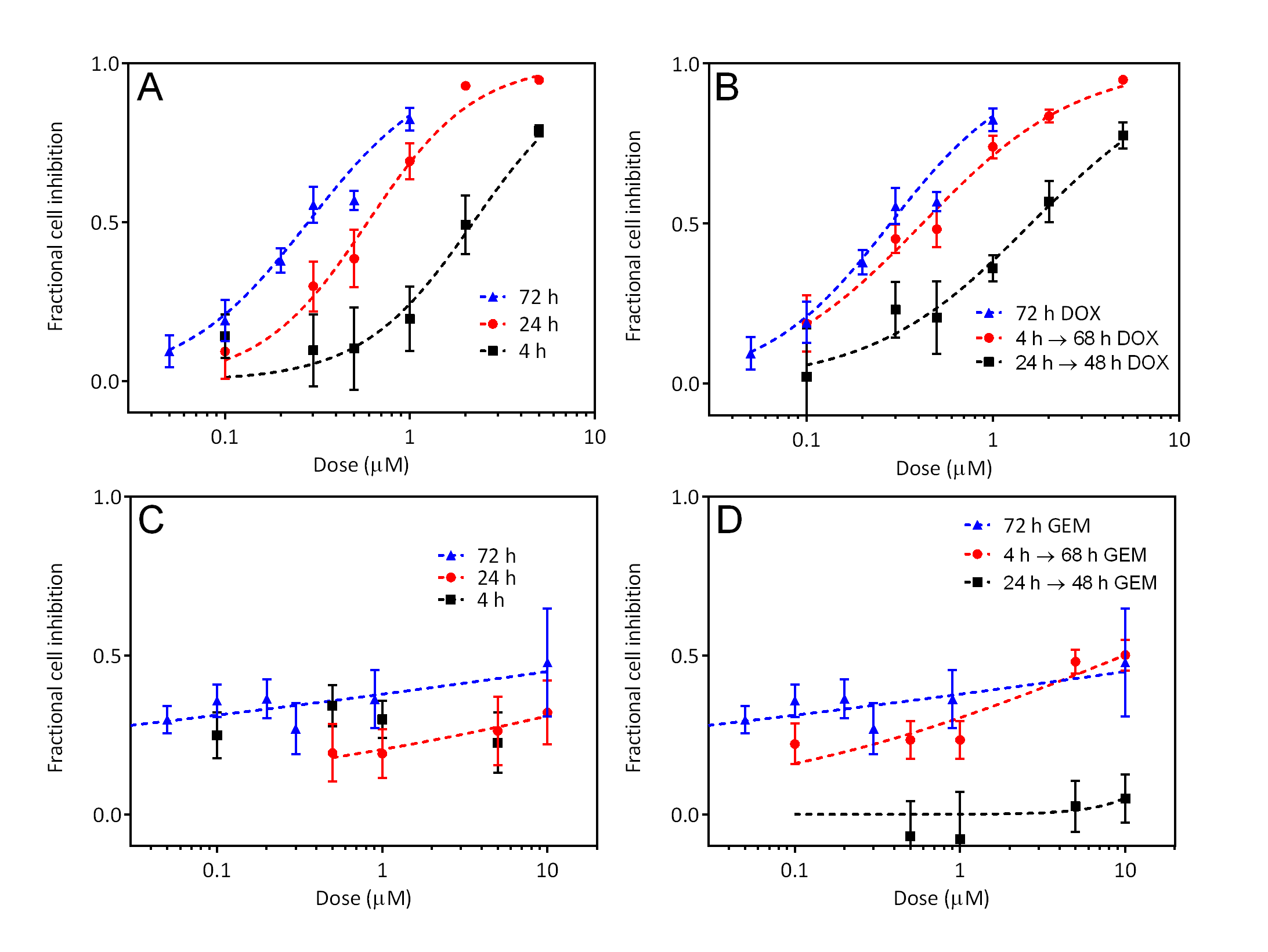


Fig. S1. Fractional cell inhibition and relevant dose response curves for MDA-MB-231 cells after exposure to (a) DOX 🡪 media (b) media 🡪 DOX (c) GEM 🡪 media (d) media 🡪 GEM. Cell viability was assessed at 72 h for each schedule. Error bars represent 95 % CI (n ≥ 18 wells).
